# Fosmid-Based Structure-Function Analysis Reveals Functionally Distinct Domains in the Cytoplasmic Domain of *Drosophila* Crumbs

**DOI:** 10.1534/g3.112.005074

**Published:** 2013-02-01

**Authors:** Sven Klose, David Flores-Benitez, Falko Riedel, Elisabeth Knust

**Affiliations:** Max-Planck-Institute of Molecular Cell Biology and Genetics, 01307-Dresden, Germany

**Keywords:** morphogenesis, polarity, PDZ-binding domain, FERM-binding domain

## Abstract

The evolutionarily conserved transmembrane protein Crumbs is required for epithelial polarity and morphogenesis in the embryo, control of tissue size in imaginal discs and morphogenesis of photoreceptor cells, and prevents light-dependent retinal degeneration. The small cytoplasmic domain contains two highly conserved regions, a FERM (*i.e.*, protein 4.1/ezrin/radixin/moesin)-binding and a PDZ (*i.e.*, postsynaptic density/discs large/ZO-1)-binding domain. Using a fosmid-based transgenomic approach, we analyzed the role of the two domains during invagination of the tracheae and the salivary glands in the *Drosophila* embryo. We provide data to show that the PDZ-binding domain is essential for the maintenance of cell polarity in both tissues. In contrast, in embryos expressing a Crumbs protein with an exchange of a conserved Tyrosine residue in the FERM-binding domain to an Alanine, both tissues are internalized, despite some initial defects in apical constriction, phospho-Moesin recruitment, and coordinated invagination movements. However, at later stages these embryos fail to undergo dorsal closure, germ band retraction, and head involution. In addition, frequent defects in tracheal fusion were observed. These results suggest stage and/or tissue specific binding partners. We discuss the power of this fosmid-based system for detailed structure-function analyses in comparison to the UAS/Gal4 system.

Many internal organs of multicellular organisms develop from epithelial tubes, which further differentiate to serve a variety of functions. They can be specialized for secretion, as salivary glands or the pancreas, or for respiration, forming branched networks of interconnected tubes, *e.g.*, in the lung of vertebrates and the tracheal system of arthropods (Affolter *et al.* 2009; Kerman *et al.* 2006; Warburton *et al.* 2010). Others are specialized for absorption and filtration, for example, the kidney or the Malpighian tubules, the excretory organs of vertebrates and arthropods, respectively (Denholm and Skaer 2009; Little *et al.* 2010). Several mechanisms are used to form tubular organs, including budding and invagination from an existing epithelium, oriented cell division, cavitation of a solid epithelial rod, or formation of a lumen by fusion of intracellular vesicles (reviewed in Andrew and Ewald 2010; Rodriguez-Fraticelli *et al.* 2011). Strikingly, many molecules and pathways involved in tube formation are conserved between invertebrates and vertebrates. This finding and the relatively simple organization of the fly embryo, its accessibility to high resolution *in vivo* imaging, and the availability of a large genetic toolbox has made the *Drosophila* embryo an ideal system to study the cell biological and genetic basis of tubulogenesis. In particular, studies of a simple tube, the salivary gland, and a branched tubular system, the tracheae, have provided detailed insight into the different steps of tubulogenesis and their regulation (Affolter *et al.* 2009; Baer *et al.* 2009; Maruyama and Andrew 2012; Pirraglia and Myat 2010; Schottenfeld *et al.* 2010).

Most processes during salivary gland and tracheal development take place in the absence of any cell division. This means that the final organization of the organ depends on changes in cell shape and cell size, on remodeling of junctions, and modification of apical and basolateral surface areas (reviewed in Andrew and Ewald 2010; St Johnston and Sanson 2011). After allocation of ectodermal cells to either salivary gland or tracheal cell fate, the initial morphogenetic processes common to both organs can be subdivided into three different steps: apical constriction, internalization, and elongation. Apical constriction depends on the coordinated activity of signaling molecules and components of the actin cytoskeleton. This leads to a shrinking of the actino-myosin belt and a reduction of the apical surface (reviewed in Sawyer *et al.* 2010). Internalization of cells occurs by coordinated and often patterned invagination, resulting in a small sac or pit. Once internalized, the sac expands to form a tube. Directed migration of the tube is under genetic control, which ensures the stereotypic localization and patterning of the organ. While the salivary glands stay as simple tubes, the tracheal sacs start to branch in a very precise and stereotypic pattern. Individual branches further elongate and eventually fuse at later stages (Affolter and Caussinus 2008).

The *Drosophila* ectoderm, from which salivary glands and tracheae originate, is a single-layered epithelial sheet, with a pronounced apico-basal polarity. A hallmark of epithelial cell polarity is the apical zonula adherens (ZA), a belt-like structure, characterized by accumulation of the homophilic adhesion molecule *Drosophila* E-cadherin, which is linked to the actin cytoskeleton via Armadillo, the *Drosophila* ortholog of β-catenin. Maintenance of epithelial cell polarity is crucial for proper embryonic development, and its loss results in severe morphogenetic defects and embryonic lethality. One of the key regulators of epithelial cell polarity is the evolutionarily conserved Crumbs complex. It is composed of the transmembrane protein Crumbs (Crb), which recruits the scaffolding proteins Stardust (Sdt), *D*PATJ, and *D*Lin-7. Embryos lacking Crb or Sdt fail to maintain the integrity of most of their epithelia. Cells of the epidermis, for example, are unable to establish a proper ZA, lose contacts to their neighbors, and undergo extensive apoptosis. The same happens to cells of the salivary glands, whereas the tracheae invaginate, but fall apart later, forming small vesicles with proper apicobasal polarity (Grawe *et al.* 1996; Tepass 1996; Tepass and Knust 1990).

Crb, a type I transmembrane protein, contains a large extracellular domain, composed of an array of repeats with similarity to the epidermal growth factor (EGF-like repeats), interspersed by four domains with similarity to the globular domain of laminin A. Its small cytoplasmic portion of only 37 amino acids contains two highly conserved motifs, a C-terminal, PDZ (postsynaptic density/discs large/ZO-1)-binding motif, -ERLI, which can bind the PDZ-domain of Sdt and *Drosophila* Par-6, and a FERM-binding motif, which can directly interact with the FERM (protein 4.1/ezrin/radixin/moesin)-domain of Yurt and Expanded (Ex) (Laprise *et al.* 2006; Ling *et al.* 2010) and is required to recruit Moesin to the apical membrane (Medina *et al.* 2002). Besides a role in epithelial cell polarity, *Drosophila crb* controls tissue size in imaginal discs by acting upstream of the Hippo pathway (reviewed in Boggiano and Fehon 2012; Genevet and Tapon 2011), regulates the morphogenesis of photoreceptor cells, and prevents light-dependent retinal degeneration (reviewed in Bazellieres *et al.* 2009; Bulgakova and Knust 2009). The modular organization of the Crb protein raises the question of whether individual domains serve particular functions. This idea is supported by the observation that one of the three mammalian Crb orthologs, Crb3, contains only the conserved cytoplasmic domain but lacks the typical extracellular domain with EGF- and laminin A-like repeats (Lemmers *et al.* 2004; Makarova *et al.* 2003).

Using the Gal4/UAS system (reviewed in Elliott and Brand 2008), we elucidated the function of the short cytoplasmic domain of Crb. We found that ubiquitous expression of a membrane-bound cytoplasmic domain, called Crb_intra_, suppressed the *crb* mutant embryonic phenotype to the same extend as the full-length protein. Removing the PDZ-binding motif (Crb_intraΔERLI_) completely abolished this activity (Klebes and Knust 2000; Wodarz *et al.* 1995). This result was in agreement with the observation that *crb^8F105^*, an allele encoding a mutant protein that lacks the C-terminal 23 amino acids, develops only a slightly weaker embryonic phenotype than the protein null allele *crb^11A22^* (Wodarz *et al.* 1993). Expression of UAS-*crb_intraY10A_* or UAS-*crb_intraY10AE16A_*, which carry mutations in conserved amino acid residues within the FERM-binding domain, also failed to suppress the *crb* mutant phenotype (numbering of amino acids according to Klebes and Knust 2000), starting with the Arginine residue C-terminal to the transmembrane domain (Figure 1). However, unlike UAS-*crb_intraΔERLI_*, these constructs induced polarity defects when overexpressed in wild-type embryos, similar to those observed with the full-length cytoplasmic domain (Klebes and Knust 2000). Using the same UAS-constructs, it was found that the FERM-binding domain is required to rescue invagination defects of the anlagen of the tracheae in *crb* mutant embryos (Letizia *et al.* 2011) and to activate the Salvador/Warts/Hippo pathway in otherwise wild-type wing imaginal discs (Robinson *et al.* 2010). On the other hand, a UAS-construct encoding the membrane-bound extracellular domain, which does not rescue the embryonic phenotype, could rescue the overgrowth phenotype in heads and eyes associated with loss of *crb* (Richardson and Pichaud 2010), but not the embryonic *crb* phenotype.

**Figure 1:**
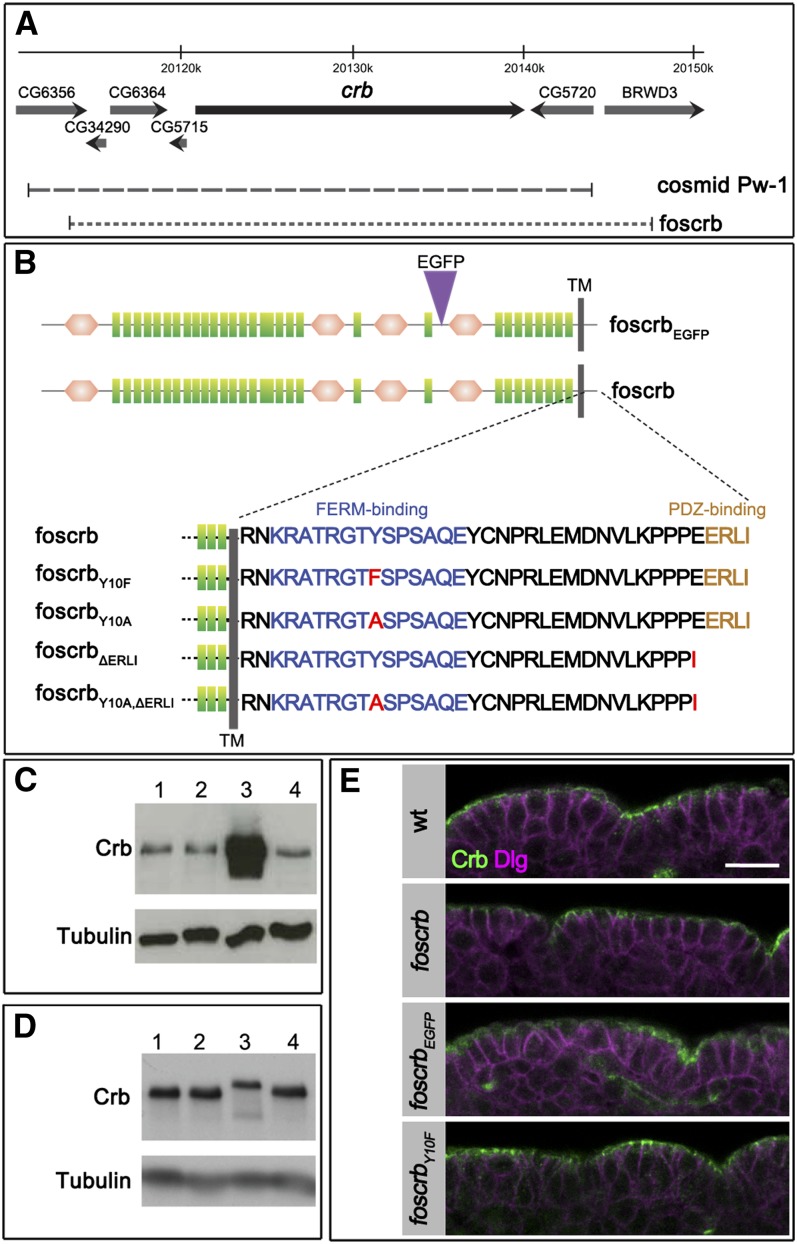
*foscrb* variants used and their expression. (A) Genomic region covering the *crb* locus (adopted from Flybase). *foscrb* (dotted line) covers nearly the same region as cosmid Pw-1 (dashed line), which was shown to rescue the *crb* mutant phenotype (Tepass and Knust 1990). (B) Crb variants used in this study. Green rectangles: EGF-like repeat, brown hexagon: repeat with similarity to the globular domain of laminin A, gray bar: transmembrane (TM) domain, purple: insertion of EGFP. Depicted proteins are based on the Crb-PA isoform (2.146 amino acids). Numbering of amino acids in the cytoplasmic domain starts with the first arginine residue (R) after the transmembrane domain. The fosmids encode all Crb isoforms, although only Crb-PA is shown here. The amino acid sequences of the wild-type and mutant versions of full-length Crb are indicated, red symbols indicate exchanges. (C) *foscrb* does not cause Crb overexpression in the presence of endogenous Crb (lane 2), whereas UAS/Gal4-driven *crb* does. Western blot of lysates prepared from overnight embryos, probed with anti-Crb and anti-Tubulin as a loading control. Embryos, from which extracts were prepared, had the following genotypes: lane 1: wild-type; lane 2: *foscrb/foscrb* (carry four copies of *crb*); lane 3: *UAS-crb_full-length_*; *GAL4^daG32^*; lane 4: *foscrb/foscrb*; *crb^GX24^/crb^GX24^*. (D) *crb*, *foscrb*, *foscrb_EGFP_*, and *foscrb_Y10F_* express Crb protein at similar levels. Western blot was probed with anti-Crb and anti-Tubulin, antibodies. Extracts were prepared from overnight embryo collections from wild-type (lane 1) and homozygous *foscrb/foscrb*; *crb^GX24^/crb^GX24^* (lane 2); *foscrb_EGFP_/foscrb_EGFP_*; *crb^GX24^/crb^GX24^* (lane 3); and *foscrb_Y10F_/foscrb_Y10F_*; *crb^GX24^/crb^GX24^* (lane 4) embryos. (E) Cross section of the embryonic epidermis at stage 13/14 showing the localization of Crb and the lateral marker Dlg in wild-type (wt) and homozygous *foscrb/foscrb*; *crb^GX24^/crb^GX24^*, *foscrb_EGFP_/foscrb_EGFP_*; *crb^GX24^/crb^GX24^* and *foscrb_Y10F_/foscrb_Y10F_*; *crb^GX24^/crb^GX24^* embryos. Scale bar, 10 µm.

Despite the tremendous power of the Gal4/UAS system, there are several disadvantages. In most cases, a heterologous promoter is used, which does not reflect the endogenous expression pattern of the gene and often results in ectopic and/or strong overexpression. In addition, only one isoform of the gene of interest is expressed. Recently developed methods using large genomic fragments, such as bacterial artificial chromosomes (BACs) or fosmids, which cover whole genes, including all splice variants and regulatory elements, overcome most of these problems (reviewed in Ejsmont *et al.* 2011; Venken and Bellen 2012). In combination with recombineering, which allows the introduction of mutations into the transgenes by homologous recombination in bacteria before insertion into the genome (reviewed in Ciotta *et al.* 2011), this technology now opens the possibility for structure-function analysis under optimized *in vivo* conditions.

Here, we used fosmid-based transgenesis to analyze the role of the PDZ- and FERM-binding domains of the cytoplasmic tail of Crb during early stages of salivary gland and tracheal development. We show that the PDZ-binding motif is essential for proper invagination of both salivary glands and tracheae. Surprisingly, however, and in contrast to previous results obtained with UAS-constructs, a Crb protein with a mutated FERM-binding domain (fosCrb_Y10A_) rescued apical constriction, invagination, and elongation of salivary glands and tracheae despite some defects during apical constriction observed during tracheal morphogenesis. Embryos expressing fosCrb_Y10A_ showed later defects, such as incomplete tracheal fusion, defective dorsal closure, and germ band retraction.

## Materials and Methods

### Fly stocks

Flies were kept at 25°. The following stocks/mutant alleles were used: OregonR as wild-type control, *crb^11A22^* (Jürgens *et al.* 1984), *crb^GX24^* (Huang *et al.* 2009), *Gal4^daG32^*, *Gal4^385.3^*, UAS*-crb_intramyc2b_* (Wodarz *et al.* 1995), UAS*-crb_8xMycintra16.1_* (S. Özüyaman and E. Knust, unpublished data), *w*; *foscrb;crb^GX24^*, *w*; *foscrb_EGFP_*; *crb^GX24^*, *w*; *foscrb_Y10F_*; *crb^GX24^*, *w*; *foscrb_Y10A_;crb^GX24^*, *w*; *foscrb_ΔERLI_*; *crb^GX24^*, and *w*; *foscrb_Y10A,ΔERLI_*; *crb^GX24^* (this work). Mutant stocks were balanced over *TM3*, *twist-GAL4*, UAS*-EGFP* (Bloomington Stock Center).

### Recombineering protocol to generate *foscrb* variants

The *foscrb* variants are based on the fosmid library clone pFlyFos No P52 G02 obtained from Pavel Tomancak [MPI-CBG, Dresden (Ejsmont *et al.* 2009); named *foscrb* throughout the text]. The contained genomic region of *crumbs* was modified by recombineering in *Escherichia coli in vivo* by use of the Red/ET Recombination technology according to the technical protocol for the “Counter-Selection BAC Modification Kit by Red/ET Recombination” (version 3, 2007; Gene Bridges) with following major changes: The recombineering as well as the amplification of the vector *foscrb* were performed in the *E. coli* strain TOP10 (Invitrogen). Whenever *foscrb* was kept in liquid culture, 0.01% L-arabinose and 20 µg/mL chloramphenicol are added (Ejsmont *et al.* 2009). The concentration of streptomycin in the counter-selection step was increased from 50 µg/mL to 8000 µg/mL to enhance the efficiency of the counter-selection. In addition, the counter-selection by streptomycin was performed overnight in liquid culture, and then 1 µL of this liquid culture was plated onto LB-Agar plates containing chloramphenicol and streptomycin for a final counter-selection step. Cotransformed recombineering source served the plasmid pRed4Flp (Sarov *et al.* 2006), whereas 0.35% L-rhamnose was used to induce Red expression and was selected by adding 100 µg/mL hygromycin in low-salt LB medium, pH 8, cultured at 30° due to the temperature-sensitive origin of replication. However, after we added L-rhamnose and before the addition of the recombineering cassette, the temperature was shifted to 37° to obtain an optimal Red expression. The plasmid pR6K-rpsL-neo (Wang *et al.* 2006) was used as template to amplify the respective counter-selection cassette in the first recombineering step. After the first as well as second recombineering step a medium-scale plasmid DNA isolation (QIAGEN), followed by retransformation into *E. coli* TOP10 cells, was performed to identify the clone containing the correct integration of the counter-selection/modification cassette by colony-polymerase chain reaction (PCR). A detailed description of the protocol can be obtained from the authors.

### Generation of *foscrb* variants

*foscrb* contains the nonmodified wild-type *crb* locus. For the generation of *foscrb_EGFP_* the oligonucleotides 5′-GGGTCAGGTGGTTCTGGCATGGTGAGCAAGGGCGAGGAGC-3 and 5′-CCCGGATCCTCCCGAGCCCTTGTACAGCTCGTCCATGCCGA-3′ were used to amplify the sequence encoding the EGFP tag from the plasmid pEGFP-C1 (Clontech), without STOP codon and to flank the tag with a stretch of glycines and serines (GSGGSG). The linker shall enhance a proper folding and reduce effects onto the Crb protein. The PCR product served as template in a second PCR to add the homology arms for recombineering using the oligonucleotides 5′-*TCGAATTTTGCCAACACGTTACATGTCCGGGACAGAGCTTGTGCCAAAAT*GGGTCAGGTGGTTCTGGCATGGTGAGCAAGGGCGAGGAGC-3′ and 5′- *TCCTGCCCAGTAAATGTGGTGTTCGTAACACACTCATAGCCATCGTCCAG*CCCGGATCCTCCCGAGCCCTTGTACAGCTCGTCCATGCCGA-3′ (*homology arms*, linker GSGGSG). The counter-selection cassette was amplified from the template pR6K-rpsL-neo (Wang *et al.* 2006) by using the oligonucleotides 5′-*TCGAATTTTGCCAACACGTTACATGTCCGGGACAGAGCTTGTGCCAAAAT*GGCCTGGTGATGATGGCGGGATCG-3′ and 5′- *TCCTGCCCAGTAAATGTGGTGTTCGTAACACACTCATAGCCATCGTCCAG*TCAGAAGAACTCGTCAAGAAGG-3′ (*homology arms*). For all *foscrb* variants mutated in the cytoplasmic domain, the same counter-selection cassette was amplified from the template pR6K-rpsL-neo (Wang *et al.* 2006) by using the oligonucleotides 5′-*GACATTGCCATCATTGTAATACCCGTAGTGGTGGTGCTGCTGCTGATCGC*GGCCTGGTGATGATGGCGGGATCG-3′ and 5′-*TGTAAACCATAACTAGGGGCCAACTTAGTACAAAACATTGAGTTACTCCT*TCAGAAGAACTCGTCAAGAAGG-3′ (*homology arms*). The oligonucleotides 5′-GACATTGCCATCATTGTAATA-3 and 5′-TGTAAACCATAACTAGGGGCCA-3′ were used to amplify the mutated cytoplasmic domains of *crumbs* from the following pre-existing plasmids: *foscrb_Y10F_* from pBS-8xMyc-crbintra-Y10F (C. Clemens and E. Knust, unpublished data); *foscrb_Y10A_* from pUAST-*8xMyc-crb_intra-Y10A_* (S. Özüyaman and E. Knust, unpublished data); *foscrb_ΔERLI_* from pBS-*8xMyc-crb_intra-ΔERLI_* (S. Özüyaman and E. Knust, unpublished data); *foscrb_Y10A_*_,_ *_ΔERLI_* from pUAST-*8xMyc-crb_intra-Y10A,ΔERLI_* (C. Clemens and E. Knust, unpublished data). The obtained modification cassettes exchanged the counter-selection cassettes in the second recombineering step in the different *foscrb* variants, respectively. All *foscrb* variants were verified by sequencing before injection into *Drosophila melanogaster*.

### Generation of transgenic flies

Transgenic flies were generated via the phiC31 integrase mediated site-specific integration into *attP* landing-sites (reviewed in Venken and Bellen 2007). For the injection and establishment of transgenic lines standard protocols were followed (Bachmann *et al.* 2008). All *foscrb* variants were integrated into the landing site *attP40*, of the stock *y*, *v*, *P(nos-phiC31\int.NLS)X* ; *P(CaryP)attP40* (Bloomington #25709).

### Embryo collection, antibody staining, and cuticle preparation

Embryos were collected on apple juice plates for 2 hr at 25° and then incubated for 6 hr at 25° or 12 hr at 19°, dechorionated in 50% bleach for 3 min, and fixed for 20 min in 4% formaldehyde in phosphate-buffered saline/heptane. For heat fixation, dechorionated embryos were sunk into boiling TTS solution (68 mM NaCl, 0.03% Triton X-100) and then transferred immediately to ice. Devitellinization was done in heptane/methanol. Embryos were blocked for 2 hr at room temperature in PBT (phosphate-buffered saline + 0.1% Triton X-100) + 5% normal horse serum. Embryos were incubated for 2 hr at room temperature with primary antibodies: rat anti-Crb 2.8, 1:500, (Richard *et al.* 2006a), mouse anti-Crb-Cq4, 1:300 (Tepass *et al.* 1990), mouse anti-Discs large (Dlg) 4F3, 1:50 [Developmental Studies Hybridoma Bank (DSHB), 1:50], mouse anti-Armadillo N2 7A2, 1:50 (DSHB) (Riggleman *et al.* 1990), rabbit anti-Stranded at second (Sas, 1:500; kindly provided by E. Organ and D. Cavener), rabbit anti-Canoe (Cno), 1:1.000 (Matsuo *et al.* 1999, kindly provided by K. Takahashi), rabbit anti-Pyd, 1:5.000 (Djiane *et al.* 2011, kindly provided by Sarah Bray), guinea pig anti-Eyegone (1:1000) (Aldaz *et al.* 2003, kindly provided by Natalia Azpiazu), rabbit anti-phospho moesin (Cell Signaling Technology, cat. no. 3150, 1:100), mouse anti-alpha-spectrin SA9, 1:25 (DSHB), rabbit anti-GFP (Invitrogen, cat. no. A11122, 1:500). Incubations with the appropriate secondary antibodies were performed for 1 hr at room temperature: 1:500 for Alexa Fluor 488-, 568-, and 647-conjugated antibodies (Invitrogen). Stained embryos were mounted in glycerin propyl gallate (75% glycerol, 50 mg/mL propyl gallate) and visualized using a Zeiss LSM 780 NLO confocal microscope with a C-Apochromat 40x/1.2W Corr objective with the correction collar at 0.18 (at this position the brightness and contrast was enhanced). All images were taken under the same settings for laser power, PMT gain and offset. Maximal projections and merging was performed using Fiji and Adobe Photoshop CS4. Cuticle preparations were performed according to standard procedures (Wieschaus and Nüsslein-Volhard 1986).

### Viability test

Adult flies of the desired genotype were kept on apple juice agar at 25° and removed after 2 hr. The number of embryos on the plate was counted and the plate was further incubated at 25°. After approximately 48 hr, the number of empty eggshells was determined and divided by the total number of embryos to determine the viability. This experiment was done three times for each genotype. In summary 201, 296, and 351 embryos were collected after 2 hr for WT; 322, 246, and 331 for *foscrb/foscrb*; *crb^GX24^/crb^GX24^*, 114, 187, and 273 for *foscrb_EGFP_/foscrb_EGFP_*; *crb^GX24^/crb^GX24^*; and 187, 175, and 169 for *foscrb_Y10F_/foscrb_Y10F_*; *crb^GX24^/crb^GX24^*.

### Preparation of embryonic extracts and western blot

For embryo collection, adult flies of the respective genotype were kept overnight at 25° on apple juice agar plates. Embryos were dechorionated for approximately 3 min in 3% bleach and homogenized with Biovortexer (Biospec products) in lysis buffer (50 mM Tris, pH 8.0; 150 mM NaCl; 0.5% Triton X-100; 1 mM MgCl_2_) supplemented with complete protease inhibitor cocktail (Roche). The lysate was centrifuged for 2 min at 4° at 1.900 g and protein concentration was determined by standard Bradford biochemistry using Roti-Quant (Roth). The supernatant was analyzed by western blotting using standard procedures. Primary antibodies used were: rat anti-Crb 2.8 (1:1.000) (Richard *et al.* 2006a) and mouse anti-α-Tubulin (1:3.000, Sigma-Aldrich).

## Results

### *foscrb*, *foscrb_EGFP_*, and *foscrb_Y10F_* completely rescue *crb*-induced embryonic lethality

*Drosophila* Crb is involved in several processes, such as maintenance of epithelial cell polarity, regulation of the Hippo pathway, morphogenesis of photoreceptor cells, and prevention of light-dependent retinal degeneration. To better understand which region(s) of this multidomain protein are required for these different functions, transgenes encoding the whole genomic region of *crb* have been designed. It has previously been shown that the cosmid clone Pw-1, containing a genomic region of approximately 32 kb, completely rescues the *crb* mutant embryonic and adult eye phenotypes [(Tepass *et al.* 1990); M. Richard and E. Knust, unpublished data]. Beside the ∼19-kb transcribed region, this clone contains ∼9 kb upstream genomic sequence of the *crb* locus and ∼4 kb downstream (Figure 1A). The fly fosmid clone *pFlyfoscrb* (no.P52GD2) used here (called *foscrb*) contains the complete transcribed region of the *crb* locus plus ∼7 kb upstream and ∼5 kb downstream genomic sequence, thus spanning a similar genomic region to that contained in cosmid Pw-1 (Figure 1A). A second variant, *foscrb_EGFP_*, was generated by recombineering, which contains an EGFP tag N-terminal to the fourth laminin A G domain-like repeat (Figure 1B), thus allowing to distinguish the transgene-encoded from the endogenous Crb protein. *foscrb* was the template for several mutant variants, which are summarized in Figure 1B. The design of these variants was based on previous constructs, which used the GAL4/UAS system (Klebes and Knust 2000; Wodarz *et al.* 1995). The deletion of the C-terminal amino acids in *foscrb_ΔERLI_* removes the PDZ-binding motif, ERLI, which links Crb with Sdt (Bachmann *et al.* 2001; Hong *et al.* 2001) and *D*mPar-6 (Kempkens *et al.* 2006). Two variants, *foscrb_Y10F_* and *foscrb_Y10A_*, carry mutations in Tyrosine_10_ of the cytoplasmic domain, which is conserved in all Crb variants described so far (Richard *et al.* 2006b). This residue is part of a conserved FERM-binding domain (Klebes and Knust 2000), which has been shown to bind the FERM protein Yurt, a negative regulator of Crb (Laprise *et al.* 2006), and the FERM domain of Ex, an upstream regulator of the Hippo pathway (Ling *et al.* 2010). In addition, a version carrying both mutations, *foscrb_Y10A,ΔERLI_*, was generated (Figure 1B).

Both *foscrb* and *foscrb_EGFP_* rescued *crb* the loss-of-function mutation *crb^GX24^* (Huang *et al.* 2009) or *crb^11A22^*: 87% and 75% of the embryos homozygous for *crb^GX24^* and carrying two copies of the fosmid, *i.e.*, *foscrb*; *crb^GX24^* or *foscrb_EGFP_*; *crb^GX24^*, respectively, hatched, which is slightly less than wild-type embryos (95%). Flies with either genotype were fertile, did not show any obvious mutant phenotype, and could be kept as homozygous stocks. In contrast, a UAS-transgene encoding the full-length Crb protein was unable to rescue embryonic lethality and could only suppress some aspects of the *crb* mutant embryonic phenotype (Wodarz *et al.* 1995). Surprisingly, 82% of the embryos with the genotype *foscrb_Y10F_*; *crb^GX24^* hatched and gave rise to adult viable and fertile flies. This result is striking because expression of UAS*-crb_intraY10F_*, which encodes a protein consisting of the transmembrane and the cytoplasmic domain of Crb, in which Tyr_10_ was mutated to Phenylalanine, did not rescue embryonic lethality and showed only minor suppression of the *crb* mutant embryonic cuticle phenotype upon ubiquitous expression, compared to that of UAS*-crb_intra_* (C. Clemens and E. Knust, unpublished data).

It is well established that the amount of Crb protein expressed in a cell is crucial for the maintenance of apicobasal polarity and proper size of the apical domain (Hamaratoglu *et al.* 2009; Klebes and Knust 2000; Muschalik and Knust 2011; Wodarz *et al.* 1995). Therefore, we analyzed the levels of Crb protein in flies carrying different copy numbers of the endogenous and/or fosmid-encoded *crb* gene. Surprisingly, the presence of four copies of *crb* did not increase the overall Crb protein levels. In the absence of endogenous *crb*, each of the three fosmids that rescued embryonic lethality expressed comparable amounts of Crb protein when present in two copies (Figure 1, C and D).

### *foscrb* and *foscrb_EGFP_* show wild-type expression pattern and subcellular localization of the Crb protein

**Figure 2:**
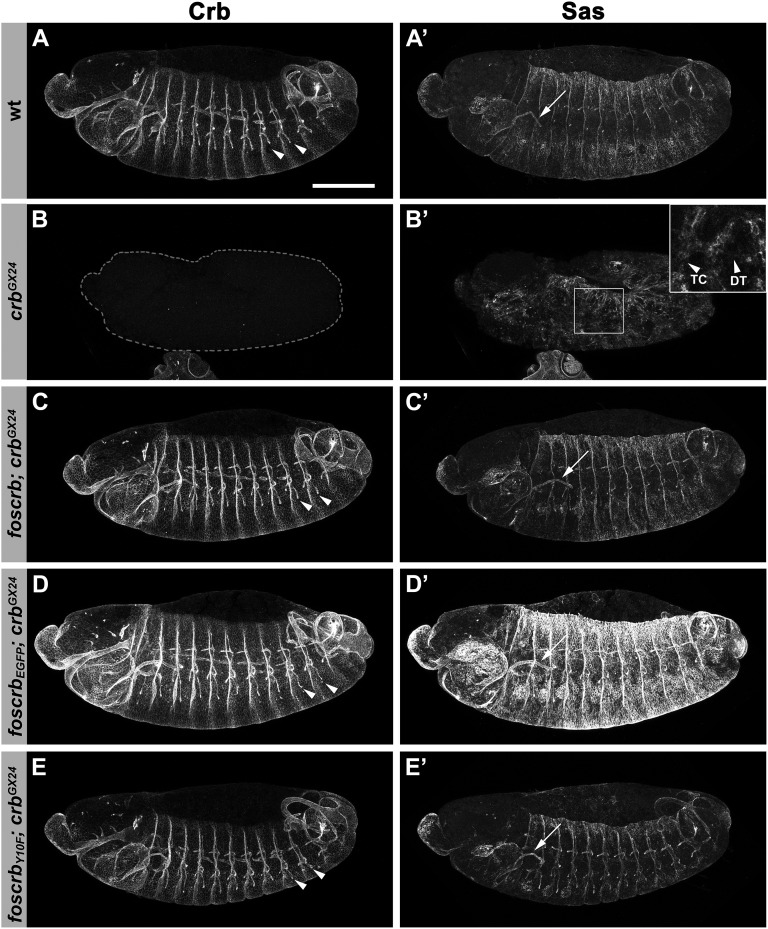
*foscrb* variants completely rescuing *crb* mutant embryos. Lateral view of stage 13/14 whole-mount embryos stained for Crb and Sas (right). (A) Wild-type. (B) *crb^GX24^*. (C) *foscrb*; *crb^GX24^*. (D) *foscrb_EGFP_*; *crb^GX24^*. (E) *foscrb_Y10F_*; *crb^GX24^*. Arrows in A′, C′, D′, and E′ indicate salivary glands, and arrowheads in A, C, D, E the tracheae. In B, the embryo negative for Crb staining is outlined with the dotted line. Inset in B′ is a maximal projection of a stack through the remnant tracheal system. The arrowheads in the inset in B′ indicate discontinuities in the transverse connective (TC) and dorsal trunk (DT) branches. Anterior is to the left, dorsal up. Scale bar, 100 µm.

To analyze the expression pattern of *foscrb*- and *foscrb_EGFP_*-encoded Crb protein in embryos in the absence of endogenous *crb*, we stained *foscrb*; *crb^GX24^*, *foscrb_EGFP_*; *crb^GX24^* and *foscrb_Y10F_*; *crb^GX24^* embryos with different markers at different developmental stages. At all developmental stages, fosCrb protein in these embryos was expressed in epithelia of ectodermal origin as in wild-type, *i.e.*, in the epidermis, the amnioserosa, the tracheae, the salivary gland, the hindgut and the Malpighian tubules (Figure 2 and data not shown). As revealed by the apical marker Sas, epithelia in embryos carrying one of these fosmids maintain proper apicobasal polarity (Figure 2, C′−E′). In embryos with these genotypes, fosCrb is localized apical to the basolateral marker Dlg (Figure 1E and data not shown).

### *foscrb_Y10A_*, *foscrb_ΔERLI_*, and *foscrb_Y10A_*_,_ *_ΔERLI_* do not rescue *crb*-induced embryonic lethality

**Figure 3:**
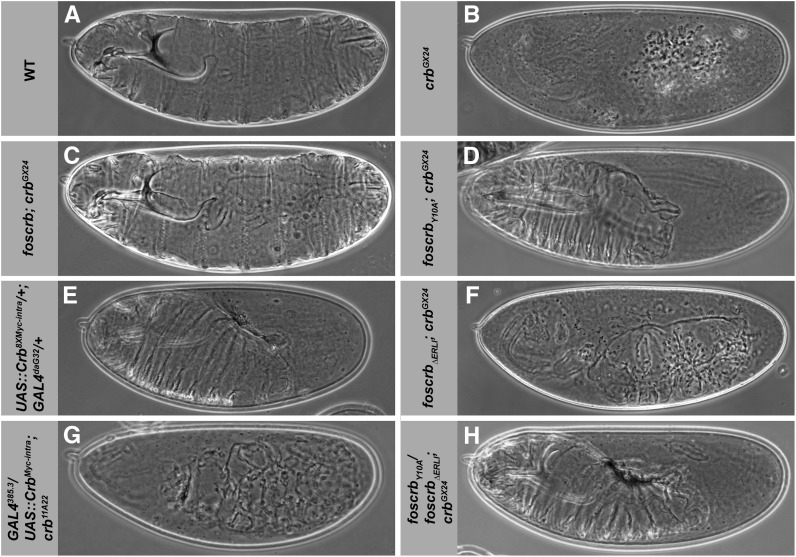
*foscrb* variants and their ability to rescue the *crb* mutant phenotype. Cuticle of *Drosophila* embryos (lateral views) with the following genotypes: (A) wild-type (WT), (B) *crb^GX24^*, (C) *foscrb*; *crb^GX24^*, (D) *foscrb_Y10A_*; *crb^GX24^*, (E) Wild-type embryo overexpressing UAS-*crb_8xMyc-intra_* under the control of a strong, ubiquitously expressed Gal4 line, *GAL4^daG32^*, (F) *foscrb_ΔERLI_*; *crb^GX24^*, (G) *crb^11A22^* mutant embryo overexpressing UAS-*crb_Myc-intra_* under the control of a ubiquitously expressed Gal4 line, *GAL4^385.3^*, and (H) *foscrb_Y10A_/foscrb_ΔERLI_*; *crb^GX24^*. Anterior is to the left, dorsal up.

*crb* mutant embryos lack a continuous cuticle, and only grains of cuticle can be detected (compare Figure 3, A and B) (Jürgens *et al.* 1984; Tepass and Knust 1990). *crb* mutant embryos with transgenes that either carried a mutated FERM-binding domain (*foscrb_Y10A_*; *crb^GX24^*), lacked the PDZ-binding motif (*foscrb_ΔERLI_*; *crb^GX24^*), or carried both mutations (*foscrb_Y10A,ΔERLI_*; *crb^GX24^*) did not hatch. The different transgenes suppressed the *crb* mutant phenotype to different degrees. In comparison with *foscrb*, which completely rescued the cuticle phenotype (Figure 3C), *crb* mutant embryos carrying two copies of *foscrb_Y10A_* form continuous anterior and ventral cuticle with rather fully developed denticle belts (Figure 3D). This result is striking in view of previous observations showing that Gal4-mediated expression of UAS-*crb_intraY10A_* completely failed to suppress the *crb* mutant cuticle phenotype (Klebes and Knust 2000). The phenotype of *foscrb_Y10A_*; *crb^GX24^* embryos resembles that of wild-type embryos overexpressing UAS-*crb_intra_* with a ubiquitously expressed GAL4 line (compare Figure 3, D and E) (Wodarz *et al.* 1995) and is characteristic for embryos with impaired germ band retraction and dorsal closure. *crb* mutant embryos carrying *foscrb_ΔERLI_* developed only small patches of continuous cuticle (Figure 3F) reminiscent to the phenotype of *crb* mutant embryos with a strong intermediate phenotype (Tepass and Knust 1990) but more severe than a *crb* mutant embryo expressing the membrane-bound intracellular domain (UAS-*crb_intra_*) using an ubiquitously expressed GAL4 line (Figure 3G) (Klebes and Knust 2000). The phenotype of *crb* mutant embryos with one copy of *foscrb_Y10A_* and one copy of *foscrb_ΔERLI_* was the same as that of *crb* mutant embryos with two copies of *foscrb_Y10A_* (compare Figure 3, D and H). *crb* mutant embryos carrying *foscrb_Y10A,ΔERLI_* did not develop any continuous cuticle and resembled *crb* embryos with a strong loss of function allele (data not shown).

Loss of *crb* differentially affects various ectodermally derived embryonic epithelia. Although some epithelia, such as the epidermis, almost completely die, others, such as the tracheal system, partially survive and their cells form vesicles that maintain epithelial cell polarity. Some organs, such as the hindgut, are nearly unaffected (Tepass and Knust 1990). To analyze in more detail the effects of the expression of the different *foscrb* variants during embryonic development, we stained these embryos for Crb and the apical marker Sas at different developmental stages.

**Figure 4:**
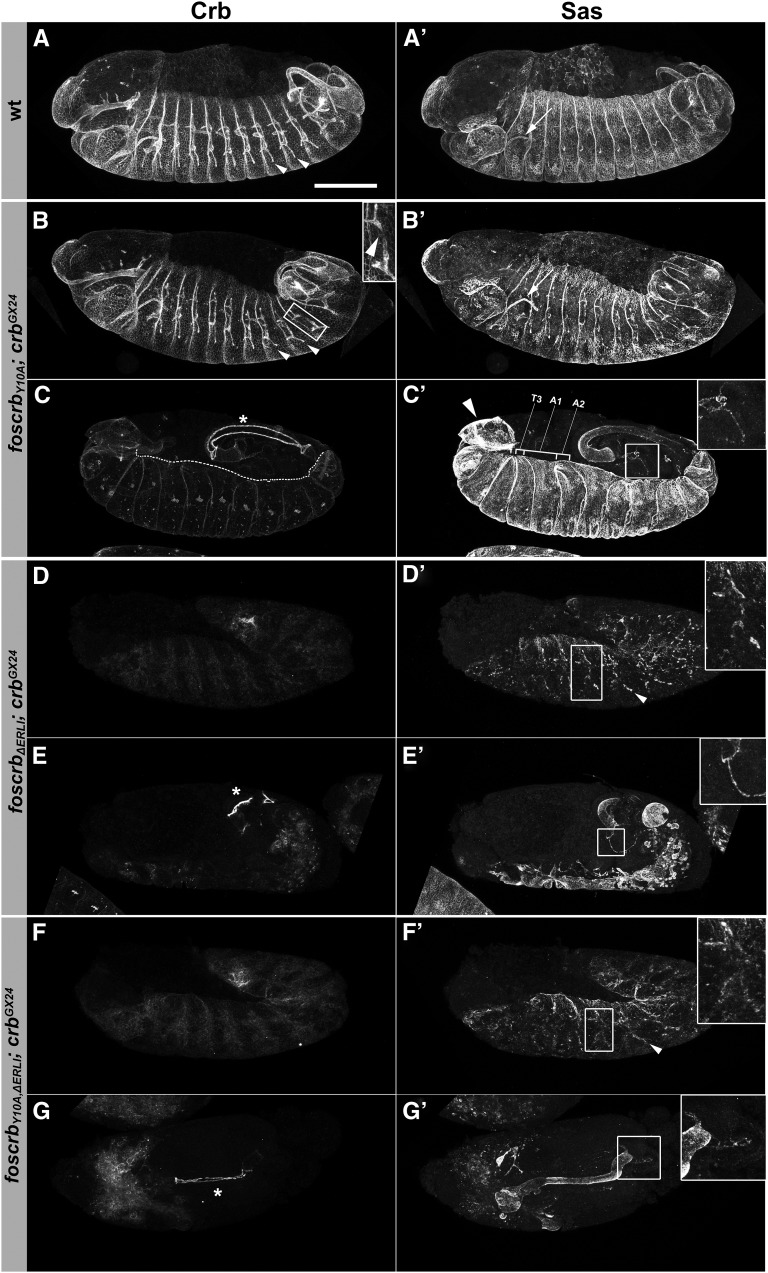
*foscrb* variants and their ability to rescue the *crb* mutant phenotype. Lateral view of whole embryos stained for Crb (left column) and the apical marker Sas (right column). (A, A′) Wild-type embryo at stage 13/14. All ectodermally derived epithelia show apical localization of Crb and Sas. (B−C′) *foscrb_Y10A_*; *crb^GX24^* embryos at stage 13 (B, B′) and 15/16 (C, C′). The arrowhead in the inset in (B) indicates a discontinuity in Crb staining along the transverse connective branch. The dotted line in (C) delineates the leading edge of the epidermis. Arrowhead in (C′) indicates the head that fails to undergo involution. In (C′), the third thoracic segment (T3) and the first two abdominal segments (A1, A2) are marked to highlight their variable width. (D-E′) *foscrb_ΔERLI_*; *crb^GX24^* embryo at stage 13 (D, D′) and stage 15/16 (E, E′). (F-G′) *foscrb_Y10A,ΔERLI_*; *crb^GX24^* embryo at stage 13 (F, F′) and stage 15/16 embryo (G, G′). Arrowheads in A, B, D′, and F′ indicate the tracheae, insets in B, D′, and F′ show tracheal hemisegments. Insets in C′, E′, and G′ show a Malpighian tubule. * in C, E, and G point to the hindgut. Arrows in A′ and B′ point to the salivary gland. Anterior is to the left, dorsal up. Scale bar, 100 µm.

Epithelial polarity and integrity in *foscrb_Y10A_*; *crb^GX24^* was normal in the epidermis (compare Figure 4, A and B), the fore- and hindgut, the salivary glands (compare Figure 4, A′ and B′, arrows), and the Malpighian tubules until the end of embryogenesis. In contrast, the amnioserosa exhibited defects from stage 13 onwards (not shown) and was completely lost at stage 15. This defect was accompanied by impaired germ band retraction, head involution (arrowhead in Figure 4C′), and dorsal closure. In fact, the leading edge of the epidermis appears uneven and wiggling, instead of straight as observed during dorsal closure in wild-type embryos (highlighted by a dotted line in Figure 4C). In addition, segments exhibited variable widths. For example, the width of segments T3, A1, and A2 is largely unequal, contrary to what is observed in wild-type embryos (Figure 4C′). This finding suggests defects in the tension of the actin cable in the leading edge cells. In contrast, the hindgut (Figure 4C, asterisk) and the Malpighian tubules (Figure 4C′, inset) were maintained. Interruption of Crb staining in some transverse connective branches manifested defects in the tracheal system already at stage 13 (Figure 4B, arrowhead in inset; see below). These discontinuities were also observed along the dorsal trunk at later stages (not shown). In *foscrb_ΔERLI_*; *crb^GX24^* embryos at stage 13, nearly no Crb protein was detected (Figure 4D). Sas expression indicated the presence of remnants of the dorsal epidermis at this stage, whereas the tracheal tubes were fragmented (Figure 4D′, inset). At the end of embryogenesis, only the hindgut (Figure 4E, asterisk), the Malpighian tubules (Figure 4E′, inset) and vesicular structures, probably remnants of the tracheae, were visible (Figure 4E′). The phenotype of *foscrb_Y10A,ΔERLI_*; *crb^GX24^* embryos was similar to that of *crb* mutant embryos carrying the *foscrb_ΔERLI_* transgene (Figure 4, F−G′).

**Figure 5:**
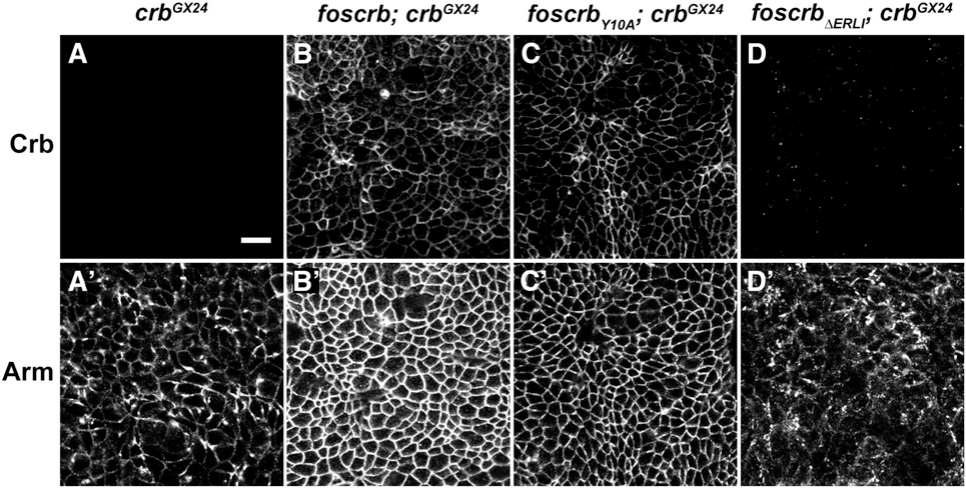
*foscrb* variants and their ability to rescue the ZA defects of *crb* mutant embryos. Optical sections taken from the epidermis of stage 11 embryos of the following genotypes: *crb^GX24^* (A, A′), *foscrb*; *crb^GX24^* (B, B′), *foscrb_Y10A_*; *crb^GX24^* (C, C′), and *forscb_ΔERLI_*; *crb^GX24^* (D, D′). The embryos were stained with Crb (A, B, C, D) and Armadillo (Arm; A′, B′, C′, D′), a component of the *zonula adherens*. Scale bar, 10 µm.

As previously shown, cells of the developing epidermis of *crb* mutant embryos fail to form a proper *zonula adherens* (ZA) (Figure 5, A and A′) (Grawe *et al.* 1996; Tepass 1996). To analyze the effects of the different transgenes on the development of the ZA we stained stage 11 embryos with antibodies against Armadillo, the *Drosophila* ortholog of β-catenin. Both *foscrb* and *foscrb_Y10A_* restored wild-type Armadillo staining, suggesting a normal formation of the ZA (Figure 5, B, B′, C, and C′), whereas *foscrb_ΔERLI_* completely failed to restore a continuous adhesion belt (Figure 5D′).

Taken together, a Crb protein lacking the PDZ-binding motif is unable to maintain epithelial polarity from early stages onwards. In contrast, the Y10A mutation in the FERM-binding domain had no effect on epithelial polarity. Embryos expressing this mutant Crb protein showed defects in fusion of tracheal tubes and in the development of the amnioserosa, resulting in defective dorsal closure, head involution and germ band retraction.

### Role of the PDZ-binding and the FERM-binding domain during tracheal morphogenesis

The *Drosophila* tracheal system originates from groups of about 40 ectodermal cells each, called tracheal placodes. They form on the lateral side of the embryo from the second thoracic segment to the eighth abdominal segment. Invagination of epithelial cells of the placode is initiated by localized apical cell constriction, which is preceded by apical enrichment of Crb and actinomyosin (Brodu and Casanova 2006; Letizia *et al.* 2011; Llimargas and Casanova 1999). At early stage 11, tracheal cells are internalized and undergo their last round of postblastodermal division. Together, apical constriction, cell rearrangements, and oriented cell divisions are important for organized invagination of tracheal cells (Brodu and Casanova 2006; Nishimura *et al.* 2007). This results in the formation of the segmentally arranged tracheal sacs or pits at stage 11, each composed of about 80 cells, which are organized in a polarized epithelial monolayer. At stage 12, expression of the chemoattractant fibroblast growth factor induces the formation of primary branches in the tracheal sac in a stereotypic pattern. These branches elongate by cell intercalation, a process that involves changing neighbors and remodeling of adhesive contacts, to form long, thin tubes. At stage 15, fusion of branches is initiated, which finally leads to an elaborate network of interconnected tracheal tubes (reviewed in Affolter and Caussinus 2008).

**Figure 6:**
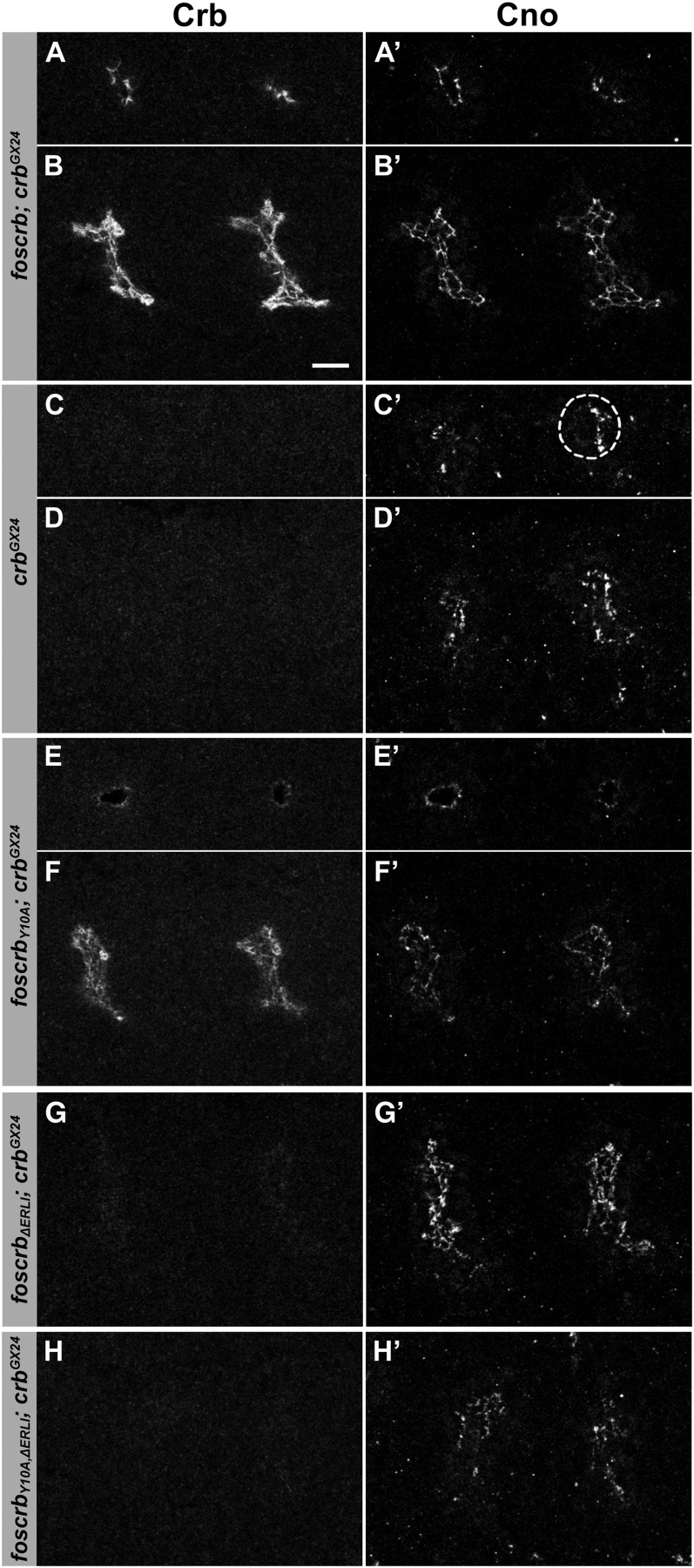
*foscrb* variants and their ability to rescue tracheal development of *crb* mutant embryos. Tracheal sacs of the second and third hemisegments of stage 11/12 embryos, stained for Crb and Cno, with the following genotypes: (A, A′, B, B′) *foscrb*; *crb^GX24^*, (C, C′, D, D′) *crb^GX24^*, (E, E′, F, F′) *foscrb_Y10A_*; *crb^GX24^*, (G, G′) *foscrb_ΔERLI_*; *crb^GX24^*, and (H, H′) *foscrb_Y10A,ΔERLI_*; *crb^GX24^*. The images shown in (A), (A′), (C), (C′), (E), and (E′) are single planes through the spiracular branch, the tube that connects the sac to the exterior. This plane of section allows one to detect whether a normal tube is formed and whether the tube develops normal apical-basal polarity. In (C′), the branch in the third segment is encircled by a dotted line. Scale bar, 10 µm.

Tracheal development was completely normal in *foscrb;crb^GX24^*, *foscrb_EGFP_;crb^GX24^* and *foscrb_Y10F_;crb^GX24^* embryos (Figure 2, C−E′, Figure 6, A and B′, and data not shown). *crb* mutant embryos, which do not express any Crb protein (Figure 2B, Figure 6, C and D), showed aberrations already in the initial invagination process at early stage 11, as revealed by the mislocalization of the ZA marker Cno, the *Drosophila* ortholog of mammalian Afadin (Figure 6, C′ and D′). Nevertheless, some internalization occurred, resulting in the formation of an irregular tracheal sac (Figure 6D′). At stage 13/14, the tracheae showed abnormal branching, discontinuities in different branches, or lack of a discernible single lumen later on (Figure 2B′, inset). At the end of embryogenesis, a complete breakdown of the tracheal system was observed (data not shown) (Tepass and Knust 1990).

Strikingly, *crb* mutant embryos expressing a Crb variant with a mutated FERM-binding domain (*foscrb_Y10A_*; *crb^GX24^*) showed nearly normal accumulation of Crb in the placode and normal apical constriction and invagination (Figure 6, E and F′). However, at stage 13, some transverse connective tubes showed discontinuities in Crb staining as in *crb* mutants (Figure 4B, inset). In later stages, gaps also were observed in the dorsal trunk (data not shown). In contrast, fosmids encoding a Crb protein, which lacked the PDZ-binding motif, completely failed to rescue the *crb* mutant phenotype in the tracheae (Figure 4, D′ and F′, Figure 6, G−H′). Similar to *crb* mutant embryos, development of the trachea in *foscrb_ΔERLI_*; *crb^GX24^* and *foscrb_Y10A,ΔERLI_*; *crb^GX24^* mutant embryos proceeded to stage 13, at which the dorsal and visceral branches as well as the posterior lateral trunk could be identified, (Figure 4D′ and F′, insets). Later on, the complete tracheal system disintegrated (Figure 4, E′ and G′).

**Figure 7:**
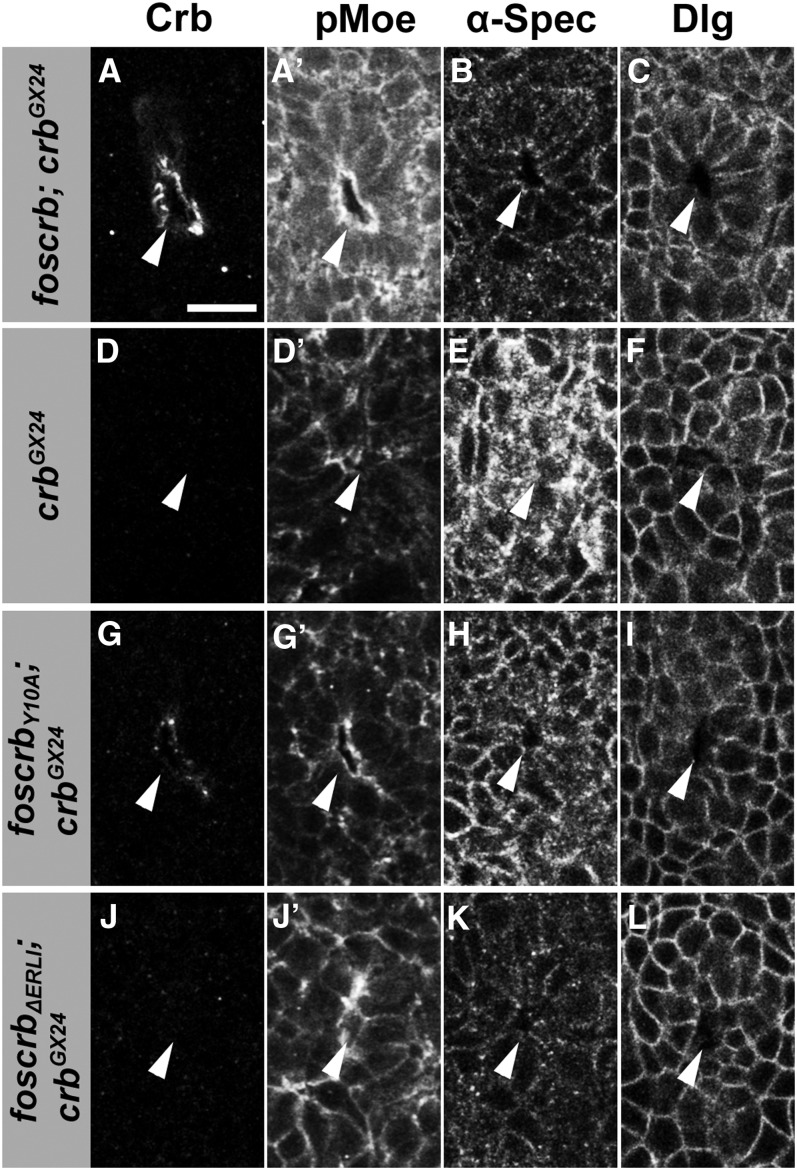
*foscrb* variants and their ability to rescue the early stages of tracheal development of *crb* mutant embryos. Spiracular branch of the embryonic tracheae at stage 11, stained for Crb, pMoe, α-spectrin (α-Spec), and Dlg in the following genotypes: (A, A′, B, C) *foscrb*; *crb^GX24^*, (D, D′, E, F) *crb^GX24^*, (G, G′, H, I) *foscrb_Y10A_*; *crb^GX24^* , and (J, J′, K, L) *foscrb_ΔERLI_*; *crb^GX24^*. The arrowheads point to the lumen observed in the reconstructed stacks of the spiracular branch. Scale bar: 10 µm.

To further dissect the early steps in the different mutant backgrounds, we used additional markers to follow the invagination process. One of the earliest events during invagination is the apical accumulation of the phosphorylated form of Moesin, phospho-Moesin, or pMoe (Letizia *et al.* 2011). Moesin is the single *Drosophila* member of the ERM (ezrin-, radixin-, moesin) protein family (McCartney and Fehon 1996). ERM proteins act as linkers between membrane proteins and the actin cytoskeleton and are crucial in organizing distinct membrane domains (Fehon *et al.* 2010). Apical pMoe, together with some apical enrichment of α-spectrin and baso-lateral localization of Dlg, highlights the apicobasal polarization of cells in the tracheal placode, both in wild-type and in *crb* embryos carrying *foscrb*, *foscrb_EGFP_*, or *foscrb_Y10F_* (Figure 7, A−C, and data not shown). Loss of polarized pMoe, α-spectrin and Dlg expression indicated loss of cell polarity in the placodes of *crb* mutant embryos (Figure 7, D−F) (Letizia *et al.* 2011). *foscrb_Y10A_;crb^GX24^* mutant embryos showed reduced levels of pMoe, diffuse staining of α-spectrin, and mostly baso-lateral, but also diffuse Dlg staining (Figure 7, G−I). These data indicate that the polarity defects of *crb* mutants were not completely rescued by the Crb_Y10A_ variant. Nevertheless, the placodes underwent normal invagination and elongation. In contrast, *foscrb_ΔERLI_*; *crb^GX24^* mutant embryos failed to accumulate pMoe and did not show polarized distribution of α-spectrin and Dlg, yet some uncoordinated internalization of cells occurred, resulting in a tiny and irregular lumen (Figure 7, J−L).

Taken together, expression of Crb_Y10A_ with a mutated FERM-binding domain allowed proper invagination of the tracheal sac and normal elongation of the branches, but was not sufficient for proper fusion of the tracheal branches at later stages. In contrast, in the absence of the PDZ-binding motif, Crb is not stabilized by Sdt, resulting in defects in apical constriction, apico-basal polarity, invagination and outgrowth of the tracheal system.

### Role of the PDZ-binding and the FERM-binding domain during salivary gland morphogenesis

**Figure 8:**
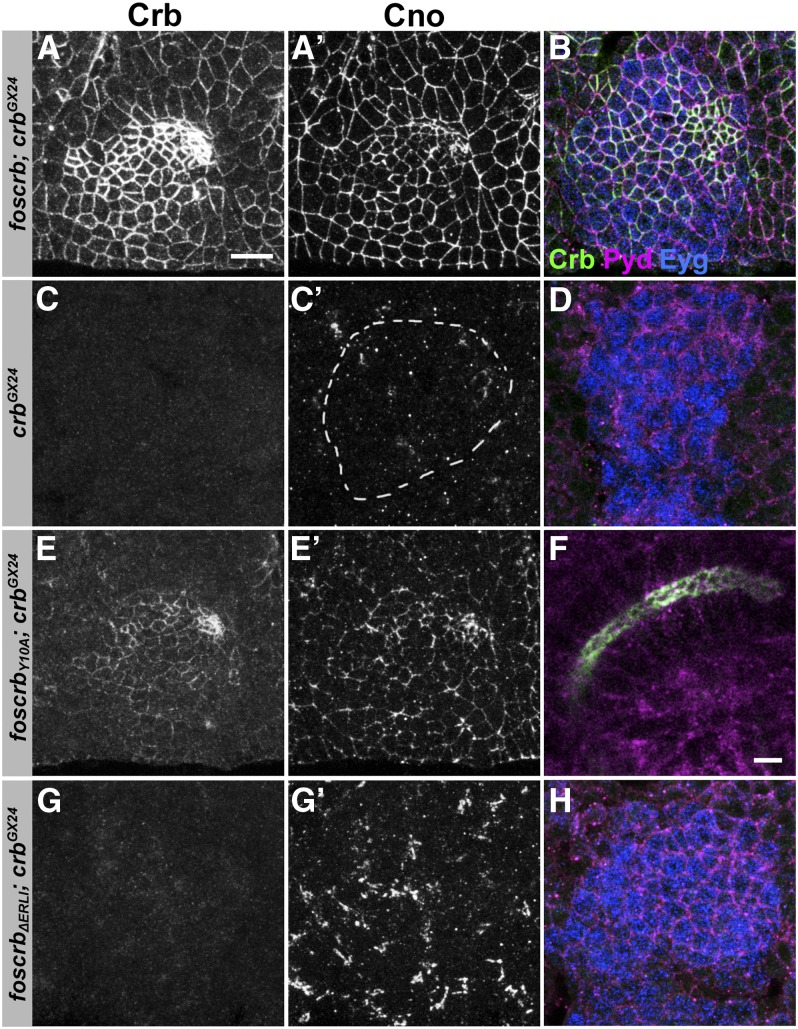
*foscrb* variants and their ability to rescue the early stages of salivary gland development of *crb* mutant embryos. Embryonic salivary gland placodes stained for Crb (A, B, C, D, E, F, G, H), Cno (A′, C′, E′, G′) Polychaetoid (Pyd; B, D, F, H, magenta) and Eyegone (Eyg; B, D, H, blue). (A), (A′), and (B) *foscrb*; *crb^GX24^* early-stage 11 embryo. (C), (C′), and (D) *crb^GX24^* stage 11 embryo. In (C′), the placode is highlighted by the dotted line. (E), (E′), and (F) *foscrb_Y10A_*; *crb^GX24^* early-stage 11 (E, E′) and stage 12 (F) embryos. (G), (G′), and (H) *foscrb_ΔERLI_*; *crb^GX24^* early-stage 11 embryo. Anti-Eyg was used in every staining to identify the salivary gland placode but only shown in B, D, F, H. Scale bar in (A),10 µm, applies to all images except for F (5 µm).

Development of the salivary glands is initiated by the formation of two placodes of approximately 100 cells each on both sides of the ventral part of parasegment two. Invagination is initiated in an orchestrated manner at stage 11, with dorsal−posterior cells constricting their apical surfaces first, followed by dorsal−anterior, ventral− anterior, and ventral−posterior cells (reviewed in Maruyama and Andrew 2012). In wild-type embryos, Crb accumulates in the salivary glands placode before invagination (Myat and Andrew 2002). A similar accumulation of Crb and the ZA markers Cno and Polychaetoid (Jung *et al.* 2006) as well as normal invagination of cells of the placode was observed in *foscrb;crb^GX24^*, *foscrb_EGFP_;crb^GX24^* and *foscrb_Y10F_;crb^GX24^* embryos (Figure 8, A and B, data not shown). In all cases, properly elongated salivary glands developed (Figure 2, C′−E′ arrows, data not shown). In *crb* mutant embryos the expression of *eyegone*, a marker for the anlage of the salivary glands (Jones *et al.* 1998), is normal, demonstrating that the salivary gland placodes are properly specified. However, cells fail to undergo apical constriction (Figure 8, C and D) and invagination does not occur. Interestingly, although *foscrb_Y10A_* partially restored accumulation of Crb in the placode (Figure 8E), apical constriction was less well organized as revealed by Cno staining (Figure 8E′). Nevertheless, invagination and elongation proceeded in a similar way as in wild-type, and Crb_Y10A_ protein was properly localized apically (Figure 8F and Figure 4B). In contrast, *foscrb_ΔERLI_;crb^GX24^* (Figure 8, G−H) as well as *foscrb_Y10A,ΔERLI_*; *crb^GX24^* (data not shown) mutant embryos did not show any apical constriction nor invagination. Similarly, and in contrast to recently published results (Roper 2012), we observed that salivary glands of *crb^8F105^* mutant embryos, which carry a premature stop codon that removes the last 23 amino acids of Crb, including the -ERLI motif (Wodarz *et al.* 1993), do not accumulate Crb and fail to invaginate (data not shown).

To summarize, *foscrb_Y10A_* allowed normal invagination and morphogenesis of the salivary gland in the absence of endogenous Crb, despite uncoordinated apical constriction at early stages. In contrast, the PDZ-binding motif of Crb is essential for all steps of salivary gland development that require Crb.

## Discussion

Two functional domains have been characterized in the cytoplasmic tail of Crb: the C-terminal PDZ-binding motif -ERLI and the juxtamembrane FERM-binding domain. Using fosmid-based transgenes, we could confirm previous results showing that the PDZ-binding domain is essential for the maintenance of epithelial polarity in the early embryo and for proper invagination of cells of the tracheal and salivary placodes. The PDZ binding domain is required for recruiting Crb to the apical membrane by binding to Sdt, and embryos expressing this truncated version of Crb develop the same phenotype as those lacking *sdt* function (Bachmann *et al.* 2001; Hong *et al.* 2001; Krahn *et al.* 2010). Similarly, the *crb* allele *crb^8F105^*, which lacks the -ERLI motif, behaves like a null allele in the embryo (Wodarz *et al.* 1993). These results are in line with the observation that UAS-*crb_intraΔERLI_*, when ubiquitously expressed in a *crb* mutant embryo, did not suppress the polarity phenotype in *crb* mutant embryos despite the presence of the FERM domain (Klebes and Knust 2000; Letizia *et al.* 2011). This finding supports the view that the PDZ-binding domain is essential for maintenance of apicobasal polarity by stabilizing the Crb-associated complex members, including Crb itself, at the plasma membrane. However, since apical localization of Crb depends on its binding to Sdt via its PDZ-domain, no conclusions can be drawn for possible function(s) of the residual part of the cytoplasmic domain under these experimental conditions.

As suggested by S2 cell culture capping assays, the truncated cytoplasmic domain encoded by *crb^8F105^* still carries an intact FERM-binding domain, which could recruit Moesin and β_H_-spectrin, but failed to do so after introducing a Tyr_10_ or Arg_7_ mutation (Medina *et al.* 2002). Therefore, we assume that the Y10A mutation in *foscrb_Y10A_* abolishes the function of the FERM-binding domain of Crb also *in vivo* and conclude that the FERM-binding domain of Crb is of little importance for epithelial polarity at early embryonic stages. However, *foscrb_Y10A_*_;_ *crb^GX24^* embryos showed later defects in dorsal closure and germ band retraction. Structural analyses have revealed that Tyr_10_ in the nonpolar region of the intercellular adhesion molecule (ICAM)-2 is crucial for the interaction with the FERM domain of Radixin. An exchange of Tyr_10_ to Ala in the cytoplasmic tail of ICAM-2 resulted in a 16-fold reduction in its binding affinity to Radixin (Hamada *et al.* 2003). Therefore, we speculate that the protein encoded by *foscrb_Y10A_* fails to interact with its FERM-domain containing partner(s). One of its binding partners is Ex, a regulator of the Hippo pathway. Results on the effect of a similarly engineered *crb* gene, which carried several mutations in the FERM-binding domain (*crb^Y10AP12AE16A^*), are controversial. Although Ling *et al.* showed basolateral mislocalisation of Ex, Robinson *et al.* showed elevated level of Ex in mutant wing disc clones (Ling *et al.* 2010; Robinson *et al.* 2010). Because the Hippo pathway has not been shown to act in the embryo, a more likely partner of the FERM-binding domain of Crb in the embryo is Yurt (Laprise *et al.* 2006). In fact, phenotypes observed in *foscrb_Y10A_;crb* embryos, *e.g.*, defects in germ band retraction and dorsal closure, are similar to those of embryos lacking zygotic *yurt* activity (Hoover and Bryant 2002). Yurt binds to the FERM-binding domain of Crb and shows apical colocalization with Crb from stage 13 onwards. Complete removal of Yurt results in apical enrichment of Crb and an expansion of the apical surface (Laprise *et al.* 2006). We observed slightly lower levels of Crb and higher levels of Sas in *foscrb_Y10A_*_;_ *crb^GX24^* embryos at later stages (Figure 4C and C′). However, more detailed analysis is required to find out whether the late phenotypes of *foscrb_Y10A_*_;_ *crb^GX24^* and *yurt* mutant embryos have the same cell biological basis. Interestingly, some aspects of the mutant phenotype of *foscrb_Y10A_;crb^GX24^* embryos, such as un-coordinated apical constriction, resemble those described for human colon cancer epithelial DLD-1 cells upon RNAi-mediated knock-down of Lulu, the mammalian ortholog of Yurt. These cells fail to organize the apical circumferential actinomyosin belt and exhibited discontinuities in β-catenin staining (Nakajima and Tanoue 2011), comparable with our observations of *foscrb_Y10A_;crb^GX24^* embryos stained with the junctional marker Canoe (see Figure 8E′).

Despite a defective FERM-binding domain, Crb proteins expressed in *foscrb_Y10A_*_;_ *crb^GX24^* embryos promote tracheal and salivary gland invagination, although these processes occur in a less-coordinated manner, possibly due to reduced pMoe recruitment. In contrast, overexpression of UAS-*crb_intraY10A_* in *crb* mutant embryos did not rescue the tracheal invagination defect of *crb* mutant embryos, which was traced back to a failure in pMoe recruitment (Letizia *et al.* 2011). This discrepancy could be the result of a dominant-negative activity of the UAS-encoded protein due to overexpression, by which the normal binding partner of the FERM-binding domain of Crb is outcompeted, leading to a delayed and uncoordinated invagination. Currently we cannot explain the discontinuities in Crb staining observed in some tracheal branches in *foscrb_Y10A_*; *crb^GX24^* embryos. Fusion of tracheal branches is a complex process, which requires, among others, formation of filopodia, *D*E-cadherin-mediated cell-cell contact and regulation of associated F-actin structures (Lee *et al.* 2003; Lee and Kolodziej 2002; Tanaka-Matakatsu *et al.* 1996). Although not studied yet, any of these processes could require ERM-protein(s), the function of which may depend on an intact FERM-binding domain of Crb.

Strikingly, *foscrb_Y10F_* rescues lethality of *crb* mutant embryos, suggesting that the phosphorylation of Tyr_10_ (as predicted by NetPhos) is not important for viability, but we cannot exclude subtle defects, such as modified dynamics of internalization. Nevertheless, this finding is surprising, given the observation that UAS-*crb_intraY10F_*, when ubiquitously expressed in a *crb* mutant embryo, suppressed the mutant phenotype much less than UAS-*crb_intra_*, which encodes the wild-type cytoplasmic domain (C. Clemens and E. Knust, unpublished data).

Our results also revealed differential requirement of Crb during tracheal and salivary gland invagination. Although cells of the tracheal anlage invaginate to some extent in *crb* mutant embryos, Crb is absolutely essential for polarity, apical constriction, invagination, and survival of cells of the salivary gland placode (Tepass and Knust 1990; Xu *et al.* 2008). In the salivary gland, the FERM-binding domain is necessary for coordinated apical constriction, organized localization of the junctional markers Cno and Pyd, and invagination movements but dispensable for internalization of cells and correct elongation of the tube. Our data do not support the function of a putative negative regulator acting via the FERM-binding because we did not observe greater Crb levels at later stages nor an expanded apical domain attributed to overexpression of UAS-Crb (Myat and Andrew 2002; Wodarz *et al.* 1995).

The presence of both a FERM binding- and a PDZ binding-motif is not uncommon in transmembrane proteins and has been described, among others, for ICAM-2, syndecans (Bass and Humphries 2002; Kwon *et al.* 2012), and the immunoglobulin-like, Ca^2+^-independent cell−cell adhesion molecule nectin (Dudak *et al.* 2011; Ishiuchi and Takeichi 2012). These proteins have short cytoplasmic domains, in which a nonpolar region, flanked on both sides by basic regions, contains a conserved tyrosine residue at position 10. The Tyr residue in the putative FERM binding domains of syndecan-3 can be phosphorylated *in vitro* (Asundi and Carey 1997). In syndecan-2, Tyr_10_ and another, more C-terminal tyrosine residue is phosphorylated by the EphB2 receptor tyrosine kinase, and this phosphorylation is essential for clustering of syndecan-2 and spine formation in hippocampal neurons (Ethell *et al.* 2001). In contrast, exchange of Tyr_10_ in syndecan-1 by phenylalanine had no effect on the association of syndecan-1 with the actin cytoskeleton (Carey *et al.* 1996), suggesting that the functional importance of phosphorylation may be syndecan and/or cell-type specific.

Taken together, although the Gal4/UAS system provides an invaluable tool to analyze gene functions, fosmid- or BAC-based transgenes combined with the recombineering technology will be our preferred approach for in depth structure-function analyses of proteins in an *in vivo* system.
